# Affordability and Availability of Child Restraints in an Under-Served Population in South Africa

**DOI:** 10.3390/ijerph17061979

**Published:** 2020-03-17

**Authors:** Prasanthi Puvanachandra, Aliasgher Janmohammed, Pumla Mtambeka, Megan Prinsloo, Sebastian Van As, Margaret M. Peden

**Affiliations:** 1 The George Institute for Global Health UK, Oxford University, Oxford OX1 2BQ, UK; margie.peden@georgeinstitute.ox.ac.uk; 2 School of Public Health and Community Medicine, University of New South Wales, Sydney 2052, Australia; 3 ChildSafe South Africa, Woolworths ChildSafe Research and Educational Centre, Red Cross Children’s Hospital, Cape Town 7701, South Africa; Ali@childsafe.org.za (A.J.); Pumla@childsafe.org.za (P.M.); 4 Burden of Disease Research Unit, South African Medical Research Hospital, Tygerberg 7505, South Africa; Megan.Prinsloo@mrc.ac.za; 5 Red Cross Children’s Hospital, University of Cape Town, Cape Town 7701, South Africa; sebastian.vanas@uct.ac.za

**Keywords:** child injury, passengers, restraints, affordability, availability

## Abstract

Background: Child road traffic injuries are a major global public health problem and the issue is particularly burdensome in middle-income countries such as South Africa where injury death rates are 41 per 100,000 for under 5′s and 24.5 per 100,000 for 5–14-year-old. Despite their known effectiveness in reducing injuries amongst children, the rates of use of child restraint systems (CRS) remains low in South Africa. Little is known about barriers to child restraint use especially in low- and middle-income countries. Methods: We carried out observation studies and parent/carer surveys in 7 suburbs of Cape Town over a three month period to assess usage rates and explore the knowledge and perceptions of parents towards child restraint legislation, ownership and cost; Results: Only 7.8% of child passengers were observed to be properly restrained in a CRS with driver seatbelt use and single child occupancy being associated with higher child restraint use. 92% of survey respondents claimed to have knowledge of current child restraint legislation, however, only 32% of those parents/carers were able to correctly identify the age requirements and penalty. Reasons given for not owning a child seat included high cost and the belief that seatbelts were a suitable alternative. Conclusions: These findings indicate the need for a tighter legislation with an increased fine paired with enhanced enforcement of both adult seatbelt and child restraint use. The provision of low-cost/subsidised CRS or borrowing schemes and targeted social marketing through online fora, well baby clinics, early learning centres would be beneficial in increasing ownership and use of CRS.

## 1. Introduction

Globally, 21% of road traffic deaths occur among children aged 0–19 year [1]. Road traffic injuries (RTIs) are the leading cause of death amongst children aged 1–19 years with global rates being estimated at 10.7 per 100,000 population [2,3] Of these, 93% occur in low- and middle-income countries (LMIC) such as South Africa (SA) where the overall (all age) road traffic fatality rate is estimated to be 33.2 per 100,000 population. Children are especially vulnerable road users in SA, with road traffic crashes being the second leading cause of death for children aged 5–14 years [4]. There is a growing concern for those children being transported in vehicles as vehicle ownership increases and there is a greater reliance on public service vehicles [5,6].

The World Health Organization (WHO) has declared that the main risk factor for young children being injured as a result of a road traffic collision is the lack of or improper use of an appropriate restraint and that this risk could be significantly reduced through the use of child restraint systems (CRS) [1]. Child restraint systems, including rear- and front-facing five-point restraint car seats and booster seats, are highly effective mechanisms to prevent child passenger injuries [7,8,9]. CRS, when correctly installed and used, can be very effective with evidence showing that they reduce the need for hospitalization by 69% in the under 5 age group and the risk of death by approximately 70% for infants and by 47% to 54% for toddlers (aged 1 to 4 years) [10,11]. A child up to 4 years of age has a 50% lower risk of injury in a forward-facing child restraint and 80% lower in a rear-facing seat [12]. This compares with injury reductions of only 32% when an adult seatbelt is worn. For children aged 5–9 years, child restraints reduce injury by 52%, whereas for seat- belts alone the reduction is only 19%. For older children aged 10–14 years seatbelts reduce injury by 46% [12].

Despite this evidence CRS use rate is low in many countries; for example, the rate of use among children under 5 years of age ranged from 7.9% to 17.4% in two Mexican cities [13]. This is similar to the observed child restraint use in Ghana with a proportion of 13.2% [14]. Even in countries with high use rate, correct and appropriate restraint use can still be a problem [15]. Observational data show that children and young adults are less likely to use seatbelts than adults [16]. According to statistics from the Child Accident Prevention Foundation of South Africa, 84% of children in vehicles are not restrained and 80% of children who had been injured in collisions were not restrained [17]. A previous observation study conducted at the main gate of the Red Cross War Memorial Hospital in Cape Town in 2008 showed that only 3% of observed children were adequately restrained [18].

Comprehensive legislation which includes laws surrounding child restraint use is a critical component in a child road traffic injury prevention effort [1]. The latest World Health Organization Global Status Report on Road Safety indicates that only 33 countries have a child restraint law in place which aligns with best practice [19]. Consequently, improving child restraint usage is one of 12 performance targets recently adopted by the United Nations General Assembly. The South African law on child restraints does not satisfy best practice. The amendment to the road safety law that was introduced in 2014 mandates the use of a South African Bureau of Standards-approved child restraint for all child passengers under the age of 3 years. This does not adhere to the United Nations (UN) prescribed best practice law that requires the use of suitable child restraints for children up to the age of 12 based on age, height and weight [20]. Experience from high-income countries shows that even when a comprehensive legislation exists, high rates of use are not possible without complimentary educational programmes, increased enforcement and supportive programmes to distribute CRS such as loan incentive schemes [10]. Barriers to increased use, particularly in LMICs include the cost of the child restraint, lack of knowledge and awareness of parents and caregivers and relatively low safety standard for vehicles [15,21,22]. However, there is little empirical data to support this. To guide the development of interventions and to persuade policy makers to focus on this under-served population, studies are needed that explore these barriers. This study aims to address the current gap in the scientific literature surrounding the barriers to CRS use in a middle-income setting by determining the availability and affordability of certified child restraints amongst different populations in Cape Town. This study was conducted as part of a larger mixed method study of child restraint use in Cape Town and therefore is not exhaustive in terms of data collected and analysis. The findings reported in this article will be combined with a systematic review of child restraint usage, a quantitative analysis of hospital data in Cape Town, qualitative data from focus groups and in-depth interviews of road safety experts in Cape Town and a review of legislation, which will serve to develop a brief intervention that will be evaluated through an appropriate study.

This study was done in collaboration with the George Institute for Global Health UK, University of Oxford, the ChildSafe team at the Red Cross Children’s Hospital, the University of Cape Town and the SA Medical Research Council. Ethical approvals were obtained from both the University of Oxford and the University of Cape Town.

## 2. Materials and Methods

To gain in-depth insight into the barriers surrounding CRS use we utilized both an unobtrusive observational study and a cross sectional survey between September 2019 to November 2019 in Cape Town, South Africa.

### 2.1. Study Setting

Cape Town is the largest city of the Western Cape Province in South Africa and is home to 64% of the Western Cape’s population. It is the second most populous city in South Africa after Johannesburg. Seven suburbs across Cape Town were selected to give a good representation of ethnic groups and socio-economic status. To try and maximize response rates and increase denominators, study sites within these suburbs were purposively selected to include hospital settings and childcare facilities. Hospitals and daycare centers were approached by the research team to request permission to carry out data collection. Table 1 shows the site selection that was used for both observational studies and the survey.

### 2.2. Observational Study

For the observational study, a standardized observation form was developed with the local ChildSafe team. Any vehicle with at least one child occupant age 14 years or under (estimated based on observations) seen to be entering the car park of the study site was eligible for the study. A team of local fieldworkers were recruited based on previous connections and knowledge of ChildSafe work. They were trained using a two-step process that included theoretical and practical knowledge. The theoretical aspect involved “in-classroom” teaching sessions to explain the nature of the study, to familiarize the fieldworkers with the survey forms and study protocols and to help them identify the different types of CRS that are currently available in South Africa. Fieldworkers were also trained on how to estimate the age of children. This was followed by the practical session in the car park of the hospital to give the fieldworkers an opportunity to use the forms and practice estimating ages of children in the front/backs of vehicles. This was used as a pilot test of the form and subsequent modifications were made to make data collection easier and more accurate. Teams of two fieldworkers were allocated specific sites and time slots. Due to the high rates of crime and violence in South Africa, the protection of fieldworkers was paramount. Homicide accounts for 56% of fatal injuries amongst individuals aged 15–34 years in South Africa [23,24]. The safety of fieldworkers was therefore ensured by the local study team with hi-visibility jackets being provided and advice given on safe zones for data collection. Data were collected in time slots of two hours throughout the day from 8am to 5pm ensuring that drop-off and pick-up times at the childcare facilities was captured. No night-time data collection occurred, again to safeguard the fieldworkers from potentially dangerous situations. Fieldworkers stationed themselves where vehicles were forced to slow down near the entrance/exit barriers to centres. Both fieldworkers were instructed to observe the cars and collect data on each vehicle, verbally verifying with each other the details of CRS usage. Forms were collated at the end of each day and taken to the ChildSafe offices for data cleaning, quality checks and data capturing.

### 2.3. Survey

For the survey, a questionnaire comprising of 30 questions was developed based on previous studies and modified to take local culture and context into consideration by translating into two additional official languages spoken in the city (Afrikaans and Xhosa) together with English. Any driver who was seen to have children was approached to respond to the survey. Asking parents/carers who had lost a child because of a road traffic injury to answer the survey was felt to be too sensitive therefore any respondent who made the fieldworkers aware of such an event was excluded from the study. Similar to the observational study, local fieldworkers were trained in both “in-classroom” sessions and field work piloting. The in-classroom teaching went through each question of the survey and interview techniques to try and maximise a positive response rate. The practice session served as a pilot and based on feedback from both interviewers and interviewees, modifications were made to the questionnaire to shorten it and to reword certain questions in South African vernacular and to restructure questions which may have been construed as being too sensitive in nature (e.g., on income level). Teams of two fieldworkers were allocated to the study sites on specific days. These fieldworkers were chosen based on whether their linguistic proficiencies in speaking the local dialect and English. Data forms were collated at the end of each day and taken to ChildSafe for data cleaning, quality checks and capturing.

Quality was assured by the ChildSafe study team for both studies with random spot checks being undertaken.

Descriptive statistics were used to summarize the main findings of the observational data with Fishers Exact test and Chi-squared statistics being used to test the relationship between variables.

## 3. Results

A total of 98 surveys (58.3%) were captured during the study period, while 70 (41.7%) of the 168 participants declined. The observational study recorded 279 observations during the study period. Table 1 gives the breakdown of survey responses and total observations by study site.

### 3.1. Observational Survey Results

Across the 7 sites, a total of 279 vehicles carrying 373 children age 0–14 were observed. Just over three quarters of the vehicles carried only one child passenger (*n* = 211; 76%), with 18% carrying two children, and 7% carrying three or more children. Approximately 16% (*n* = 59) of observed children were estimated as being less than 1 year old, 41% (*n* = 154) between the ages of 1 to 3 and 42% (*n* = 160) were categorised as older than 3 years old. 306 children were observed to be seated in the back seats compared with 67 children in the front seat.

**Figure 1:**
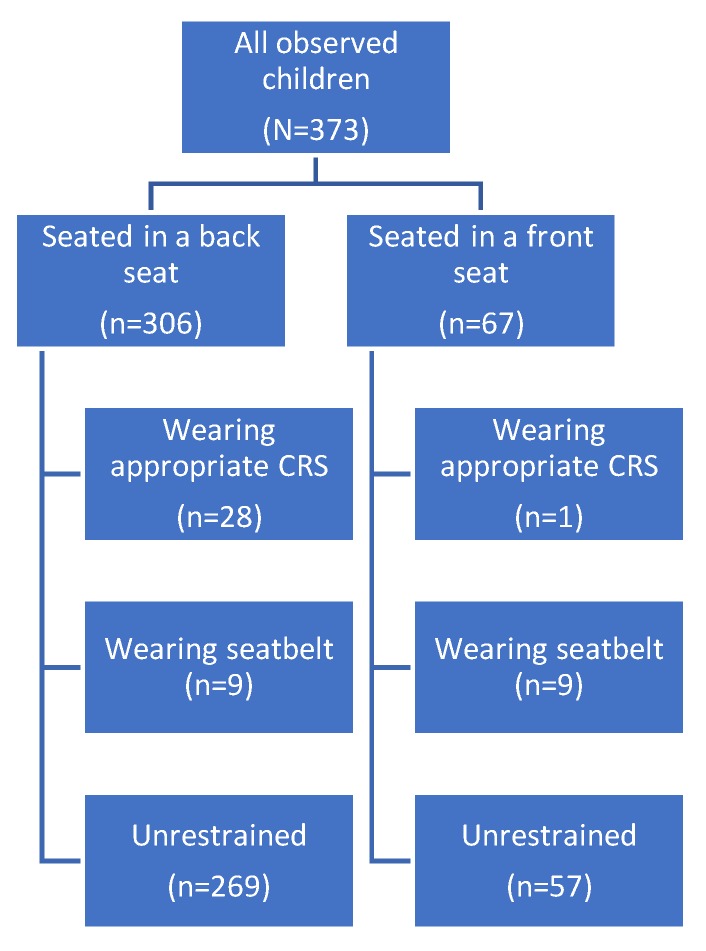
Numbers of children observed to be restrained and unrestrained in the front and back seats.

Overall, 326 children were observed to be unrestrained (87.1% 95% CI 83.6–90.6%) with only 29 children (7.8% 95% CI 5.3–11%) observed to be restrained in a formal CRS in either the front or back seat and 18 children observed to be wearing seatbelts (5.1% 95% CI 3.1–7.8%). Of those seated in the backseat, the majority (*n* = 269) were unrestrained (87.9% 95% CI 83.7–91.3%) whilst only 28 of all children seated in the back seat (9% 95% CI 6.2–13%) were observed in a formal CRS. Eleven of the 67 children who were observed to be seated in the front seat of the car were restrained with just one child using a dedicated child restraint (16.4% 95% CI 8.5–27.5%) (Figure 1). The Pearson chi-square statistics indicate that there was no significant relationship between seating position in the vehicle and child restraint use (*p*-value 0.527).

Table 2 shows the number of restrained and unrestrained child occupants stratified by age and location in the car. For all child occupants, the Fisher’s exact test statistics indicated that there was no significant relationship between age and restraint use (either seatbelt or restraint use; *p*-value 0.184).

Only 92 drivers of the 279 cars observed were seen to be wearing a seatbelt themselves (33% 95% CI 27.5–38.8%). Table 3 shows the relationship between driver seatbelt use and CRS use. Adjusting for multiple children per car with the same driver, the Pearson chi-squared statistic demonstrated that there was a significant relationship between driver’s seatbelt usage and use of restraint for child (*p*-value 0.000). 68 cars that had more than one child per car, the majority of these (*n* = 62, 91.1%) had all child occupants unrestrained with the remaining 6 cars having a mix of unrestrained and restrained children. The Fishers exact statistic indicated a significant relationship between multiple children in one car and use of child restraint (*p*-value 0.000).

### 3.2. Survey Results

#### 3.2.1. Demographics

Excluding the 24 surveys that were undertaken by the fieldworker who did not adhere to study protocol and was identified by the quality checks, a total of 168 people were approached by fieldworkers to participate in the affordability/availability survey across the 7 sites. Of these, 98 were willing to respond giving an overall response rate of 58%. Table 4 shows the summary demographic data of the survey respondents.

#### 3.2.2. Knowledge of the Current Law

**Figure 2:**
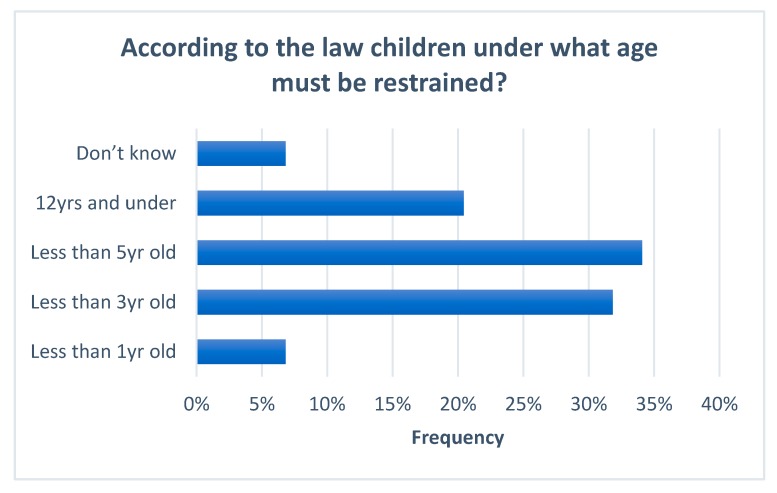
Survey respondent knowledge of current South African law on child restraints. Respondent knowledge of child restraint legislation in South Africa. (NB Correct age according to the law is “Less than 3yrs old”).

**Figure 3:**
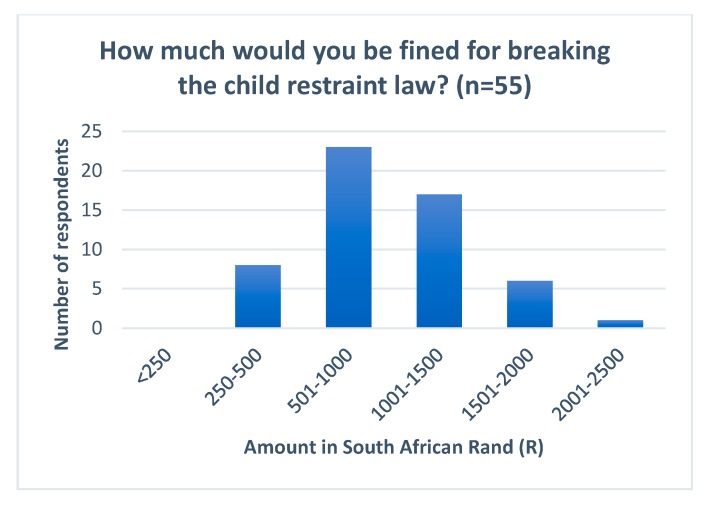
Survey respondent knowledge of fine penalty. Respondent knowledge of fine penalty for breaking the child restraint law. (NB Correct response was <250 Rand).

Ninety-two percent (*n* = 96) of respondents who answered the question about legislation claimed to have some knowledge of the legal requirements for child restraints in South Africa. However, only 32% of those respondents were able to correctly identify the age requirements stipulated in the law under which a child must be restrained (under 3 years old) (Figure 2). Seventy percent correctly identified that the police penalty for breaking the law was to receive a fine whereas 24% believed that they would only receive a warning. Those that correctly identified a fine as being the penalty received were asked to state the monetary value of the fine (Figure 3). No respondents correctly identified the legal fine of 250 R (17USD). Statistical tests showed a significant relationship between knowledge of law and education level (*p*-value 0.001) and monthly family income (*p*-value 0.000). There was no statistical difference based on gender, age of driver or years of experience in driving.

#### 3.2.3. Importance of Child Restraints

The overwhelming majority of respondents (*n* = 92; 95%) stated that they believed it was important to correctly secure a child under 3 years of age in an appropriate child restraint. Of the 5 respondents that believed it was not important, the reasons cited included that they did not feel restraints were effective, that is was safer to have the child sit on an adult’s lap, that they were too expensive, it was too difficult to install and that they were an inconvenience to use. Ninety-two percent (*n* = 82) of those who did feel it was important stated that it was because it was required by law with 83% (*n* = 74) stating that they felt it was important as they could be fined by a traffic officer if they did not use one. A further 93% (*n* = 83) believed it to be safer for the child if they used an appropriate child restraint.

#### 3.2.4. Child Restraint Ownership

**Figure 4:**
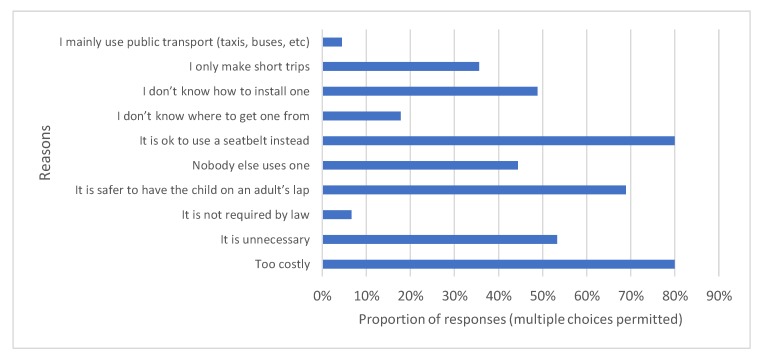
Reasons for not owning a child restraint amongst survey respondents. Reasons for not owning a child restraint system.

Of the 93 respondents who answered the question, almost half (49%) did not own a child restraint. Pearson chi-squared statistics indicated that there was a significant relationship between level of education and CRS ownership and income level and ownership. The reasons given for not owning a child restraint are shown in Figure 4 below with the majority citing costliness and a belief that seatbelts are an adequate alternative.

Those that did own a child restraint were asked to answer a series of questions regarding their purchase (Table 5). As may be expected, the Pearson chi-squared statistical tests showed a significant relationship between purchasing a new CRS from the store or online and family income level (*p*-value 0.001). Of those that owned a child restraint 36% disclosed that they did not always use the child restraint. The most common reasons stated for not using the child restraint were the belief that it was unnecessary for short trips (*n* = 10), the child did not like to sit in the seat (*n* = 10) and that the respondent owned more than one car and didn’t have child restraints in all cars (*n* = 8). Four respondents believed it was as safe to have an adult holding the child on their lap.

#### 3.2.5. Information on and Cost of Child Restraints

The majority of respondents stated that they would look online for information pertaining to CRS (88%) with a further 80% turning to magazines/books and 78% seeking advice from family and friends. A third of respondents (33%) stated that they would not be willing to pay for a child restraint and a further 22% would be willing to pay up to R500 (33USD). Twenty-four percent would be willing to pay over R1000 for a CRS (67USD).

## 4. Discussion

The observational study showed that, despite the passing of legislation in 2014, CRS use among children remains very low in Cape Town (7.8%). This is disappointingly lower than previous studies from 2008 and 2019 in South Africa which indicated rates of 11% and 18% respectively [18,25]. This proportion is significantly lower than some high-income countries where rates of 86–99% have been reported [26]. However, the prevalence is comparable to other developing countries such as Mexico, Ghana and China [13,14,27,28,29] where restraint use for children under 5 years of age is less than 4%. This is further supported by the findings from the survey and other studies which suggest that higher education levels and income levels are linked with increased CRS use and ownership [30]

A large proportion of children (84%) were observed to be sitting in the front seat of the vehicles, unrestrained. It is known that children who are unrestrained or sitting in the front seat face the greatest risk for death in road traffic crashes [31,32]. The relationship between driver seatbelt use and CRS use is well documented in the literature [33,34]. This potentially has implications in terms of opportunities to influence parental/carer seatbelt usage through increased enforcement and targeted education programs surrounding seatbelt legislation in South Africa [35].

In this study there was a significant association between CRS use and driver seatbelt use and multiple children. These findings are found elsewhere in the literature particularly in LMIC such as Nigeria and Brazil [36,37,38]. This supports the premise that if efforts to increase seatbelt use amongst adults were strengthened, it may lead to increased child restraint use if parents are more sensitized to the importance of seatbelts and restraints and if children, who are known to learn by example and replicate the actions of their parents also engage in the process.

The parent/carer survey indicated that there was a high level of reported knowledge surrounding child restraint legislation in South Africa (92%), however, when asked to accurately recall the age limits of the law only a small proportion could do so (32%). Whilst 74% of respondents correctly identified a fine as being the penalty received for breaking the law, no respondents could recall the precise fine amount with all responses estimating it to be higher than the current legal fine of 250 R (17 USD). The findings from the survey add strength to the argument that the current legislation in South Africa remains ambiguous as to the correct age that a child should be restrained and the appropriate positioning of the child [20]. It also supports the notion that the current fine remains too low to act as a deterrent to drivers in South Africa driving with unrestrained child passengers [20].

Results from the survey indicate that whilst most parents/carers (95%) believed that child restraints were important, only 51% actually owned a car seat and 36% of those who had one admitted to not always using it. The reported use was higher than in other countries such as Turkey (20%) and Kuwait (26%) [39,40]. The relatively low proportion of parents/carers who admitted to not always using a car seat despite owning one (36%) demonstrates that although parents/carers are aware of the need to use a restraint, there is a perception that there are certain circumstances where it is acceptable to not use one (e.g., on short trips, if the child does not like it, or if an adult was able to hold a child on their lap). These findings of inconsistent use are highlighted in other studies and suggest the need for targeted education interventions for such populations [41,42]. Utilising platforms such as well baby clinics, maternity wards, early learning centers to increase the general awareness of risks and debunking of certain myths surrounding CRS use would be beneficial. ChildSafe, a campaign run by the Child Accident Prevention Foundation of Southern Africa (CAPFSA) and Safe Kids Worldwide, promotes the use of CRS through organised events, workshops, targeted leaflets/posters and home safety education programs empowering parents/carers of previously injured children to prevent future such incidents. [43]. Studies from other low-income countries have shown that road safety programs which combine policy, enforcement, education and advocacy can be effective in increasing the rate of both seatbelt and child restraint usage. In Lipetskaya, child restraint usage increased from 20.9% to 51.4% from baseline to four years after program implementation [44].

Reasons given for not owning a CRS were diverse however the majority of respondents made the argument that the use of a seatbelt was an adequate alternative and reported that the cost of car seat was too high. Of those who did own a car seat, the majority of CRS had been bought new from a store (54%) or online (17%) suggesting that parents/carers were more willing to pay money for new car seats. The costs of car restraints in South Africa range from R 1000 (67USD) to R 4600 (310USD) which is more than what 55% of the respondents were willing to pay. As highlighted, there was a relationship between income level and purchasing of a new car seat with those in the lower income brackets being less likely to have purchased a new seat from a store or online. This finding is mirrored in other studies where those in lower socio-economic groups were less willing to pay for car seats [45]. The provision of low-cost/subsidised CRS or borrowing schemes would help to mitigate against lower ownership rates. Organizations such as Wheel Well have been increasing the awareness of parents about the importance of CRS and increasing use through car seat exchange programs which give low-income families the opportunity to receive a second-hand car seat in exchange for an affordable donation [46]. Given the large proportion of respondents stating that they would look online for information regarding their car seats, increasing ownership through targeted social media marketing may prove effective. Other financial motivations may include car insurance programs which provide incentives to parents who buckle up their children in appropriate CRS.

This study has several limitations. Available human, time and financial resources only permitted for a short window for data collection and therefore the sample sizes involved for both the survey and the observations is relatively low. Estimation of the age of children was also a limitation with the observational study. The fieldworkers were given some training in how to estimate such ages however, particularly with seated children being viewed through car windows, these estimates may not have been accurate. Given that the current legislation in South Africa pertained to children under 3 years of age, a decision was made to categorize children into under 3 year old and above 3 years in order to try and minimize such errors. This has implications for data analysis and its generalizability to the wider geographical area. However, this is the first such study in Cape Town which expands the site selection from a tertiary children’s hospital, which may integrate an element of bias if parents/carers felt the need to carry sick children in their arms, to include several suburbs with a mix of ethnic groups and rural/urban settings.

## 5. Conclusions

Whilst evidence-based strategies to promote seat-belt usage in LMIC have been well researched, the same is not true of child restraint usage. Whilst evidence-based strategies to promote seatbelt usage in LMIC have been well researched, the same is not true of child restraint usage and as such, this study uniquely provides essential information regarding barriers to the use of, and willingness to pay for child restraints amongst underserved populations in South Africa. The findings from this study have implications in other countries in the region and where similar disparities in income exist. It is hoped that the findings from this study and the previous qualitative studies can be used to leverage support for future research focused on implementing appropriate low-cost interventions for child injury prevention, e.g., brief interventions with high-risk mothers and/or an implementation trial of child restraints, in low = income settings. These future programs of research will generate the evidence needed to encourage policymakers to include these activities in municipal and ultimately national policies and plans.

## Figures and Tables

**Table 1:** Total number of survey responses and observed vehicles in study sites.

Site.	Suburb	Survey Responses			Observational Study
		Yes	No	Total	
**Daycares:**					
Site 1	Seapoint	22	25	47	56
Site 2	Gugulethu	4	3	7	20
Site 3	Athlone	2	8	10	21
**Hospitals:**					
Site 4	Khayelitsha	17	9	26	33
Site 5	Mitchells Plain	23	15	38	83
Site 6	Athlone	17	4	21	44
Site 7	Wynberg	13	6	19	22
**TOTAL**		98 (58.3%)	70(41.7%)	168	279

**Table 2:** Numbers of observed children stratified by age and seating position.

	Age			Total
	<1 Year	1–3 Years	>3 Years	
**Front Seat**				
Unrestrained	3	30	24	57 (85.1%)
Restrained in CRS	0	1	0	1 (1.5%)
Restrained by seatbelt	0	2	7	9 (13.4%)
Total	3	33	31	67 (100%)
**Back Seat**				
Unrestrained	49	99	121	269 (87.9%)
Restrained in CRS	7	21	0	28 (9.2%)
Restrained by seatbelt	0	1	8	9 (2.9%)
**Total**	56	121	129	306 (100%)

**Table 3:** Child restraint use compared to driver seatbelt use.

	Child in Restraint?		Total
Driver Wearing Seatbelt?	*n*	Y	
*n*	250 (73%)	7	257
Y	94 (27%)	22	116
Total	344	29	373

Chi^2^ *p*-value = 0.000.

**Table 4:** Characteristics of Survey Respondent.

Characteristic		*n*	%
Relationship to the children) in car (*n* = 98)	Mother	38	39%
	Father	49	50%
	Other Relative	6	6%
	Friend of child’s parents	3	3%
	Taxi driver/Hired Driver	2	2%
	Other	0	0%
Sex of respondent (*n* = 78)	Male	47	60%
	Female	31	40%
Age of respondent (*n* = 94)	<30 years old	14	15%
	30–39 years old	42	45%
	40–49 years old	30	32%
	50–59 years old	6	6%
	≥60 years old	2	2%
Age of child(ren) in car (*n* = 133)	0–2 years	36	27%
	3–8 years	83	62%
	9–14 years	12	9%
Highest level of education of respondent (*n* = 90)	No schooling	1	1%
	Primary school	0	0%
	Secondary or high school	48	53%
	Post school education (such as college or university)	41	46%
Number of years that respondent has been driving (*n* = 94)	<10 years	33	35%
	10–14 years	37	39%
	15–19 years	12	13%
	≥ 20 years	12	13%
Does respondent own the car? (*n* = 88)	Yes	85	97%
	No	3	3%
Total Monthly Family Income in South African Rand * (*n* = 97)	< R1 600	0	0%
	R1 600–R3 200	1	1%
	R3 201–R6 400	4	4%
	R6 401–R12 800	10	10%
	R12 801–R25 600	16	16%
	R25 601–R50,000	5	5%
	> R50,000	3	3%
	I don’t know	20	21%
	Do not wish to answer	38	39%

* At the time of manuscript preparation 1USD was equal to R14.83.

**Table 5:** Factors associated with purchase of child restraint.

	*n*	%
**Where was car seat purchased?**		
Hand me down from relative or previous child	11	23%
Garage sale	1	2%
Bought online	8	17%
New from store	26	54%
Car seat exchange program	0	0%
Gift, Brand new	2	4%
**How much did the car seat cost?**		
It was free (e.g., a gift or handed down)	11	22%
R1–R999.99	7	14%
R1000.00–R2999.99	27	53%
R3000.00–R4999.99	3	6%
R5000.00–R6999.99	1	2%
R7000.00 and above	0	0%
I Don’t know	2	4%
**What factors were important when purchasing the car seat?**		
Colour	23	14%
Price	19	12%
Style/look	24	15%
Quality	35	22%
Brand/company name	8	5%
Easy to install	23	14%
Age appropriate seat	21	13%
Recommended to me by somebody	8	5%
Other (please specify)	1	1%
